# Long non-coding RNAs in gastric cancer: New emerging biological functions and therapeutic implications

**DOI:** 10.7150/thno.47548

**Published:** 2020-07-11

**Authors:** Huidan Tan, Shouyue Zhang, Jin Zhang, Lingjuan Zhu, Yanmei Chen, Hongmei Yang, Yi Chen, Yang An, Bo Liu

**Affiliations:** 1 State Key Laboratory of Biotherapy and Cancer Center, Department of Gastrointestinal Surgery, West China Hospital, Sichuan University, Chengdu 610041, China.; 2 College of Traditional Chinese Materia Medica, Shenyang Pharmaceutical University, Shenyang 110064, China.; 3 Department of Plastic Surgery, Peking University Third Hospital, Beijing 100191, China.; 4 College of Stomatology, West China Hospital, Sichuan University, Chengdu 610041, China.

**Keywords:** Long non-coding RNA (lncRNA), Gastric cancer (GC), Biological function, Biomarker, Therapeutic application

## Abstract

Gastric cancer (GC) is currently the fourth most common malignancy and the third leading cause of cancer-related deaths worldwide. Long non-coding RNAs (lncRNAs), transcriptional products with more than 200 nucleotides, are not as well-characterized as protein-coding RNAs. Accumulating evidence has recently revealed that maladjustments of diverse lncRNAs may play key roles in multiple genetic and epigenetic phenomena in GC, affecting all aspects of cellular homeostasis, such as proliferation, migration, and stemness. However, the full extent of their functionality remains to be clarified. Considering the lack of viable biomarkers and therapeutic targets, future research should be focused on unravelling the intricate relationships between lncRNAs and GC that can be translated from bench to clinic. Here, we summarized the state-of-the-art advances in lncRNAs and their biological functions in GC, and we further discuss their potential diagnostic and therapeutic roles. We aim to shed light on the interrelationships between lncRNAs and GC with respect to their potential therapeutic applications. With better understanding of these relationships, the biological functions of lncRNAs in GC development will be exploitable, and promising new strategies developed for the prevention and treatment of GC.

## Introduction

Gastric cancer (GC), one of the most common malignant tumors, is the second leading cause of cancer-related deaths worldwide after lung cancer and is a major cause of cancer-related mortality in China 1. GC is classified anatomically into gastric adenocarcinoma and gastro-esophageal borderline adenocarcinoma. Histologically, it can be divided into two types: diffuse and intestinal 2,3. With the present surgical techniques and the implementation of traditional radiotherapy, chemotherapy, and neoadjuvant therapy, 5-year overall survival of patients with early GC can reach 95%. However, GC is seldom diagnosed early and therefore in most patients the disease has progressed to an advanced stage by the time of clinical manifestation. As a result of the malignant invasion and metastasis that is seen in later stages, the traditional treatments of surgery and chemoradiotherapy are not as effective as in early disease, and the 5-year survival rate is <20% 4. Currently, the main treatment for advanced GC (AGC) includes combinations of chemoradiotherapy, molecular targeted therapy, and immunotherapy. In order to better understand the pathogenesis of GC and identify more effective biomarkers predicting prognosis and response to treatment, extensive research is needed on all aspects of the disease. Recently, compelling evidence has emerged for an important role of long non-coding RNAs (lncRNAs) in the pathogenesis and progression of GC through intricate molecular signaling networks.

The human genome contains a vast array of nucleotides, and these complex sequences can be transcribed and finally translated into more than 100,000 proteins. Of these, only 2% constitute transcripts that encode mature proteins; the remaining 98%, called non-coding RNAs (ncRNAs), do not encode proteins but still play essential roles in cell biology 5. NcRNAs with more than 200 nucleotides are classified as lncRNAs; these account for more than 80% of ncRNAs, and are the new focus points in cancer research. Many lncRNAs have conserved secondary structures, shear forms, and subcellular localization; their conservation and specificity suggest that they are functional. However, their functions are more difficult to determine than those of microRNAs and proteins, because they cannot be inferred from their sequences or structures alone 6. Based on their locations in the genome relative to the protein-coding genes, lncRNAs can be divided into five types: sense, antisense, bidirectional, intronic, and intergenic. Compared with the transcriptional orientation of the coding RNAs, the transcriptional orientation of lncRNAs can be antisense in addition to sense. Antisense lncRNAs transcripts are initiated inside or 3' of protein-coding genes, transcribed in the opposite direction to the protein-coding genes, and overlap by at least one exon. Bidirectional lncRNAs are initiated in different ways from the promoter of a protein-coding gene; the distance that constitutes bidirectionality is usually within hundreds of base pairs. Intron lncRNAs are initiated within introns of protein-coding genes and overlap entirely with these introns. Finally, intergenic lncRNAs are defined with different transcription units from protein-coding genes 7. From another point of view, lncRNAs can be divided into nuclear lncRNAs and cytoplasmic lncRNAs, and this classification according to subcellular localization plays a key role in predicting their function. Most nuclear lncRNAs are involved in the regulation of epigenetic events and transcription, while lncRNAs in the cytoplasm play a major role at the post-transcriptional level. Thus, knowledge of such positional relationships is useful to infer the functions of lncRNAs. In addition, the promoter upstream transcripts (PROMPTs) and enhancer-associated RNAs (eRNAs) transcribed from promoters and enhancers, respectively, are similar to regulatory DNA elements in terms of their functional mechanisms 6. Unlike other lncRNAs, PROMPTs share some regulatory elements with the promoters of adjacent protein-coding genes. The expression and function of PROMPTs is often associated with adjacent protein encoding transcripts. In mammals, PROMPTs are transcribed in the antisense orientation, about 0.5-2.5 kb from the transcription start sites (TSSs) of the protein-coding gene 8. eRNAs are a group of lncRNAs transcribed from enhancers, which can promote enhancer function, and their regulation of gene expression is an emerging research field. eRNAs can recruit transcription factors, transcriptional co-activators, and chromatin remodelers in either cis or trans to regulate gene transcription and hence biological processes. Unlike common lncRNAs, eRNAs have similar transcription rates but produce fewer stable transcripts 9. In addition, lncRNAs possess certain characteristics similar to those of mRNAs; for instance, lncRNAs are transcribed by RNA polymerase II, they contain a promoter, and their structure consists of multiple exons, equipped with 5' caps and 3' poly A tails that are spliced into mature transcripts by a typical mechanism 10. Similar to the protein-coding genes, their genomic positions are marked by Histone H3 lysine K4 (H3K4) trimethylation at the transcription initiation site and H3K36 trimethylation enrichment throughout the genome; however, compared to the protein-coding transcripts, there are fewer exons in lncRNAs and the overall level of expression is lower 11. Despite being initially regarded as “transcriptional noise,” accumulating evidence indicates that lncRNAs influence local or global gene expression via transcriptional, post-transcriptional, and epigenetic regulatory mechanisms 12. Over the past decade, significant progress has been made in the study of GC-related lncRNAs, which are involved in a variety of tumor signaling pathways, such as Notch, mTOR, NF-κ B, and Wnt 13.

With the advent of high-throughput sequencing technology, novel lncRNAs, considered an important class of RNA, are being intensely investigated. Some lncRNAs have been found to have functional roles in the pathogenesis and development of cancer, and could be potential therapeutic targets or biomarkers for diagnosis and prognosis. Thus, these new findings will provide an insight into more new strategies for the diagnosis and treatment of lncRNAs in gastric cancer.

## Multi-functional roles of lncRNAs in gastric cancer

**Figure 1:**
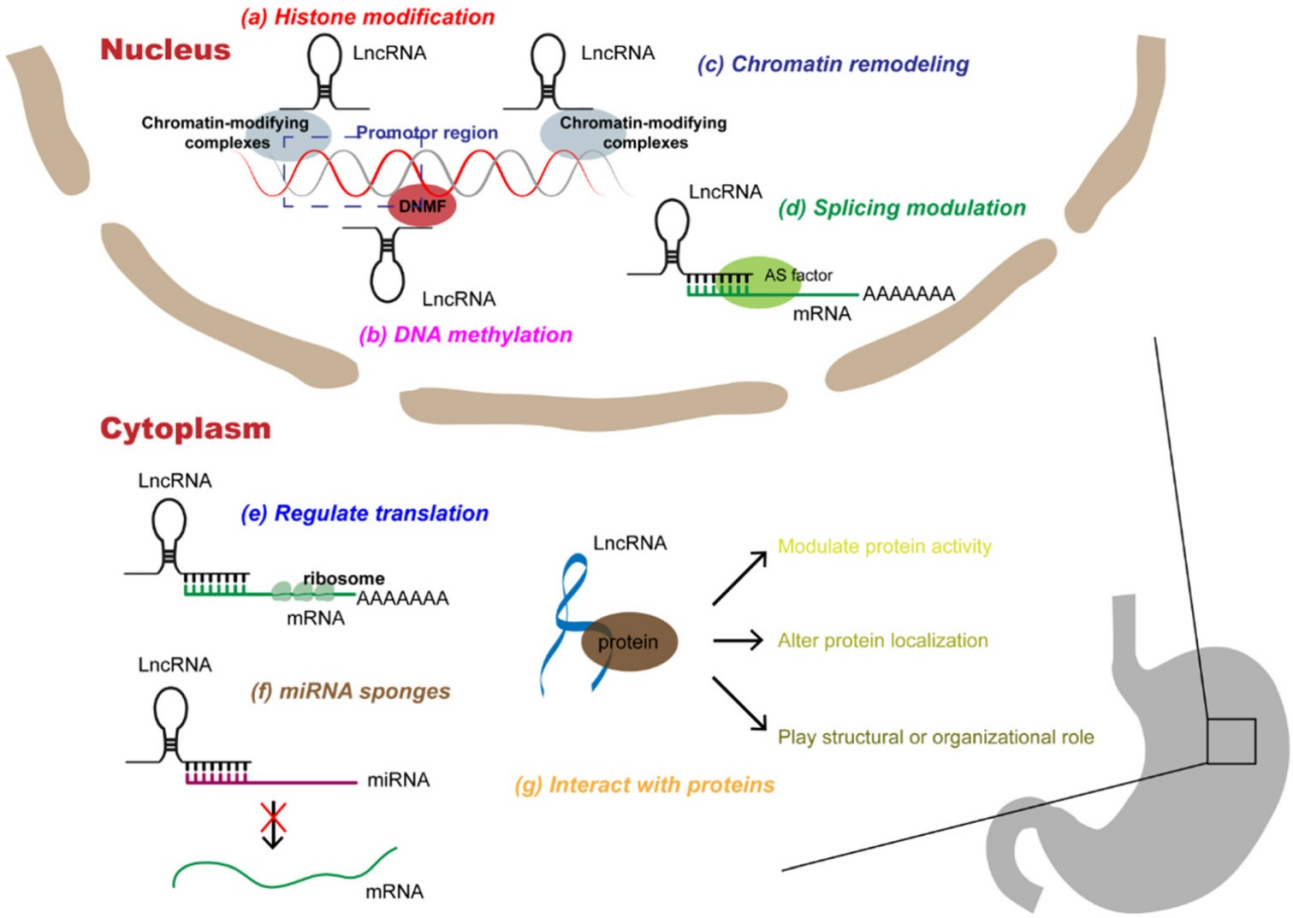
** Multi-functional roles of lncRNAs in gastric cancer.** In the nucleus, lncRNAs can participate in a variety of epigenetic mechanisms by recruiting chromatin modifiers, including **(a)** histone modifications, **(b)** DNA methylations, **(c)** chromatin remodeling; in addition, lncRNAs can participate in **(d)** splicing modulations of mRNAs. In the cytoplasm, lncRNAs can interact with RNAs, which can not only **(e)** rugulate translation of mRNAs, but also act as **(f)** miRNA sponges to alleviate miRNAs' inhibition of target genes. In addition, lncRNAs can directly **(g)** interact with proteins, affecting protein activity, localization, or as structural components.

LncRNAs are involved in many epigenetic mechanisms, including histone modification, DNA methylation, hydroxymethylation, and chromatin remodeling, which control the expression of regulatory factors involved in the progression of GC, such as genes related to DNA repair, apoptosis, autophagy, and regulators of cell cycle, transcription, and signaling pathways 14. In addition, lncRNAs can hybridize with pre-mRNAs to modulate their alternative splicing by blocking the recognition of splice sites by spliceosomes to produce alternative transcripts 15. LncRNAs located in the cytoplasm most commonly act as molecular sponges of miRNAs, thereby regulating the expression of the downstream miRNAs 16. Moreover, lncRNAs can bind directly to proteins, affecting protein activity, localization, and protein-protein interactions 17. Therefore, here we specifically describe three levels of molecular mechanisms of lncRNAs in the progression of GC, namely at the DNA, RNA, and protein levels (**Figure 1 and Table 1**).

### LncRNAs and epigenetic modifications

#### Histone modifications

Epigenetic modifiers are by far the most common protein partners of lncRNAs, and considerable attention has been lavished on their members, including histone methyltransferase and Polycomb Repressive Complex (PRC2) 18. PRC2, a methyltransferase composed of EZH2, SUZ12, and EED, epigenetically regulates gene expression by catalyzing the methylation and trimethylation of lysine residue 27 of histone 3 (H3K27me3) 19.

About 20% of human lncRNAs are reported to associate physically with PRC2, suggesting that they may play a general role in recruiting PRC2 to its target genes 20. Other epigenetic modifiers including LSD1, SMYD3, WDR5, SET1C, KMT2C, ING2, and PAQR3 may also be potential partners for lncRNAs 21-23. One of the many lncRNAs acting on epigenetic modifiers is urothelial cancer-associated 1 (UCA1). RNA sequencing (RNA-seq) analysis showed that UCA1 knockout preferentially affects genes related to the cell cycle, proliferation, and migration. UCA1 promotes cell proliferation by binding to the histone methyltransferase EZH2 to inhibit the expression of Sprouty RTK signaling antagonist 1 (SPRY1) and P21, a cyclin-dependent kinase (CDK) inhibitor 24. SPRY1, part of the mammalian sprouty gene family consisting of four members (SPRY1-4), has tumor suppressive activity and is downregulated in certain tumors such as prostate and breast cancer 25,26. UCA1 was found to mediate the trimethylation of H3K27 in P21 and SPRY1 promoters 24. Therefore, it can be concluded that UCA1, via its action on EZH2, can also influence GC progression, at least partially, through the epigenetic inhibition of P21 and SPYR1.

As an important member of the TEA domain (TEAD) family, TEAD4 is considered to be a key transcription factor involved in the development of various cancers including GC 27,28. By binding to MNX1-AS1 (MNX1 antisense RNA 1) promoter, TEAD4 facilitates the transcription of MNX1-AS1, and acts as a carcinogen at the level of transcription. MNX1-AS1 can also act on EZH2 by recruiting EZH2 and H3K27me3 to the BTG2 (B-cell translocation gene 2) promoter and thus partially inhibiting BTG2, a tumor suppressor, thereby contributing to carcinogenicity in GC 29.

The expression of Small Nucleolar RNA Host Gene 3 (SNHG3) is elevated in GC, and high SNHG3 is associated with poor prognosis 30. Because it is well-established that the mode of action of lncRNAs is by regulating adjacent genes, the relative expression of the MED18 (mediator subunit 18) gene adjacent to SNHG3 was analyzed 31. MED18, a component of the Mediator complex and coactivator of DNA-binding factors that activate transcription by RNA polymerase II, is involved in regulation of lipid and peroxisome metabolism. SNHG3 knockdown weakened the enrichment of EZH2 in the MED18 promoter, which led to a decrease in methylation level and the upregulation of the MED18 promoter 30.

RNA immunoprecipitation (RIP) experiments showed that the lncRNA ARHGAP27P1 specifically bound only to the chromatin modifier Jumonji domain-containing 3 (JMJD3). Over-expression of ARHGAP27P1 enhanced the association between ARHGAP27P1 and JMJD3, resulting in a significant decrease in H3K27me3, thus eventually facilitating the transcription of P15, P16, and P57. Furthermore, the inhibitory effect of ARHGAP27P1 on cell proliferation and cell cycle progression can be reversed by knocking down JMJD3, P15, or P16 32.

Studies have also revealed an involvement of some lncRNAs in GC through their action as modular scaffolds of WDR5 and KAT2A complexes, such as GCLNC1 and GCAWKR; some lncRNAs such as LINC00673 and FEZF1-AS1 act directly on LSD1, and thus regulate the expression of their downstream targets 21,33-35.

Increased lncRNA-AK058003 expression is frequently found in hypoxic GC. This regulates the expression of γ-synuclein (SNCG), a tumor metastasis-related gene involved in hypoxia-induced GC metastasis. AK058003 promotes hypoxia-induced GC metastasis and invasion by activating SNCG via the initiation of DNA demethylation of its CPG islands 37.

Studies on MEG3 in diabetic patients indicated that potential DNA methylation in the promoter region resulted in the reduction of target gene expression, thereby affecting disease development 38. This was also observed in GC, where MEG3 levels are significantly lower and its methylation significantly greater in tumor cells than in para-cancerous tissues, as demonstrated in multiple GC cell lines 39. Furthermore, DNA methylation affects the expression of MEG3 and its target miR-29 in HCC 40. Similarly in GC, DNA methylation affects the expression of the MEG3 target miR-181a-5p, a tumor suppressor, thus downregulating the downstream target ATG4B (autophagy-associated 4B) 39.

### LncRNAs act at the post-transcriptional level

#### mRNA translation

Some lncRNAs affect RNA translation by binding and stabilizing mRNAs. Recently, MACC1-AS1 (MACC1 antisense RNA 1), which is elevated under metabolic stress, was shown to promote the stability of MACC1 (metastasis-associated in colon cancer-1) mRNA by physically binding to it, as well as increasing its expression 41. MACC1, a key regulator of the HGF/c-MET axis, is an important targ*e*t for tumor therapy 42. In addition, MACC1-AS1 has been shown to enhance metabolic plasticity by upregulating MACC1 and subsequently enhancing glycolysis and antioxidant capacity, possibly through the AMPK/Lin28 pathway 41. AMPK and Lin28 are both important RNA-binding proteins (RBPs) that regulate the stability of mRNA.

Ephrin A1, which is either upregulated or downregulated in different cancers, is associated with tumor progression, malignancy, and prognosis 43. GC cells exhibit high amounts of GMAN (gastric cancer metastasis associated long noncoding RNA), a sense lncRNA, which competitively binds to antisense GMAN RNA (GMAN-AS), resulting in polymer formation of the downstream Ephrin A1 mRNA and thus increasing the translation of Ephrin A1 mRNA. This promotes the invasive activity and metastasis of GC cells. Knocking out GMAN or Ephrin A1 in GC cell lines reduces their invasive activity and ability to form metastases in mice 44.

#### Competitive endogenous RNA (ceRNA)

It has become increasingly clear that multiple RNA transcripts, such as ncRNAs, lncRNAs, pseudogenes, and circular RNAs, can be act as ceRNAs, also known as “molecular sponges” of miRNAs, to regulate the expression of miRNA target genes through miRNA response elements (MREs) 45. It is worth noting that a variety of lncRNAs involved in the progress of GC can be regulated in this manner, and lncRNA-miRNA-mRNA network alterations are considered indispensable mechanisms in GC. There are several examples of lncRNAs functioning as sponges and therefore oncogenes or tumor suppressor genes in GC.

KCNQ1OT1, an miR-504 sponge that targets CDK16 (cyclin‑dependent kinase 16) in HCC and CCNE2 (cyclin E2), and a miR-370 sponge, in glioma can also act as an ceRNA via neutralizing miR-9. This promotes the progression of GC by regulating the expression of Lim homeobox transcription factor 1 (LMX1A) 46,47. LMX1A is a tumor suppressor and member of the LIM-homeodomain (LIM-HD) family 48, which is downregulated in cancers 49. Moreover, in GC cells in which LMX1A is knocked out, over-expressing or silencing KCNQ1OT1 is completely ineffective, consistent with the notion that KCNQ1OT1 participates in GC progression by regulating the expression of miR-9-LMX1A 50. Another lncRNA that can control the progression of GC by regulating the miR-9-LMX1A axis is LINC00682. Ectopic over-expression of LINC00682 can downregulate miR-9 and upregulate LMX1A, as well as decrease GC cell survival, proliferation, migration, and invasion 51.

The level of expression of chemokine (CXCmotif) ligand (CXCL12) was found to be significantly increased in GC tissues with high COL1A1-014 expression. Bioinformatics analysis revealed that the two genes have complementary sequences for miR-1273H-5P; COL1A1-014 might act as a ceRNA and compete with CXCL12 to bind with miR-1273h-5p, thus inhibiting the degradation of CXCL12 by miR-1273H-5P and promoting GC progression. Additionally, BGC-823 cells over-expressing COL1A1-014 also exhibited strong colony-forming ability, which may reflect the degree of malignancy and have important significance for prognosis 52.

MYOSLID, found to be elevated in GC, is mainly concentrated in the cytoplasm 53. MCL-1 is a member of the anti-apoptotic BCL2 family that is over-expressed in many tumor types and protects tumor cells from invasion 54,55. MYOSLID promotes cell proliferation and inhibits apoptosis by regulating the miR-29C-3P-MCL-1 axis and miR-29C-3P thus acts as a tumor inhibitor in GC 53.

Neuroepithelial cell transforming gene 1 (NET1) is a RHOA (Ras homolog gene family member A) specific guanine exchange factor (GEF); RHOA is one of the most widely studied members of the RHO GTPase family of proteins. The RHOA signaling pathway is involved in biological processes such as cell proliferation and apoptosis and is related to the invasion and metastasis of tumors 56. CTC-497E21.4, involved in post-transcriptional regulation in the cytoplasm, competes with miR-22 and regulates the expression of NET1 through the RHOA signaling pathway 57. Low miR-22 expression also promotes extracellular matrix (ECM) remodeling and epithelial-to-mesenchymal transition (EMT) by enhancing matrix metalloproteinase (MMP)14 and Snail expression in GC invasion and metastasis 58.

### LncRNAs act on proteins

In addition to the above-mentioned complex regulations at the epigenetic and transcriptional levels, lncRNAs can also interact with functional proteins in the most direct manner. By regulating the activity and function of proteins, influencing the interactions between proteins, or changing the localization of proteins in cells, and even functioning as structural components, lncRNAs participate in the development of cancers.

Desmoplakin (DSP), the first discovered member of the Plakin family of proteins and a significant component of desmosomal plaques, is considered to play an important role in the early stages of tumor development 59. There is a negative correlation between MIR4435-2HG and DSP, with the former inhibiting the latter, which stimulates the growth and metastasis of GC cells by inducing EMT and Wnt/β-catenin signaling. Further, the interaction between DSP and MIR4435-2HG has been verified by RNA pull-down test, and the positive rather than the negative MIR4435-2HG pull-down protein complex was detected 60.

KRT19P3 (Keratin 19 Pseudogene 3) can regulate the activity and enhance the stability of COPS7A in GC by binding directly to it. COPS7A belongs to the CSN complex and is composed of eight subunits designated CSN1-CSN8. Abnormal expression of CSN reportedly affects key processes in carcinogenesis such as signal transduction, cell cycling, and apoptosis. COPS7A enhances de-ubiquitination of IκBα (inhibitor of nuclear factor kappa-B (NF-κB)), by the CSN-associated de-ubiquitinase USP15; thus, KRT19P3 suppresses the NF-κB signaling pathway in a COPS7A-dependent manner 61,62. As a consequence, decreased expression of KRT19P3 is associated with tumor size, TNM staging, Lauren grade, lymph node metastasis, and poor prognosis.

LINC00707, 3087 bp long, was previously reported to be an important oncogene in lung adenocarcinoma 63. Recently, it has been found that LINC00707, which also functions as an oncogene in GC, can interact with HuR, which itself interacts with 3′UTR regions of mRNA after transcription and enhances its stability; the resulting LINC00707-HuR can further combine with VAV3/F11R mRNAs to increase their stability 64,65. There is abundant evidence that VAV3 plays an important role in cell adhesion, angiogenesis, and cell differentiation, and F11R, also known as junctional adhesion molecule A (JAM-A), significantly influences epithelial cell morphology and migration 66,67.

In addition to HuR, RNA-binding protein YBX1 (Y-box-binding protein 1) also participates in the regulation of lncRNAs in GC. YBX1 is a protein with a common nucleic acid-binding domain in the gene promoter CCAA T-box, which influences many cell processes, including transcription and translation 68. HOXC-AS3 potentially regulates a group of genes related to cell proliferation and migration of GC, including MMP7, WNT10B, HDAC5 and others, by binding to YBX1 69. Among these, HDAC5 is a key gene belonging to the histone deacetylase (HDAC) family. Because histone acetylation and deacetylation are essential for chromatin remodeling and gene expression, imbalances in these reactions lead to disorderly expression of many cancer-related genes 70. In addition to HDAC5, numerous genes related to tumorigenesis are also regulated in a similar manner by HOXC-AS3 69.

## Relationships between lncRNAs and the hallmarks of gastric cancer

GC carcinogenesis is a multi-step process influenced by many different factors, such as heredity and environment. A variety of complex cancers share several common characteristics, as identified by Hanahan and Weinberg, including sustaining proliferation signals, avoidance of growth inhibitors, resistance to cell death, replicative immortality, induction of angiogenesis, activation of invasion, and metastasis 96. Each factor contributes to the formation of large numbers of abnormal cells that grow unchecked. In recent years, the exploration and elucidation of the structures and functions of lncRNAs have shown these to be important molecular signal transduction media in cancer development in that they regulate the expression of specific genes and corresponding signal pathways. Below, we will discuss the regulatory role of some lncRNAs in GC (**Table 2**).

### LncRNAs and proliferation

**Figure 2:**
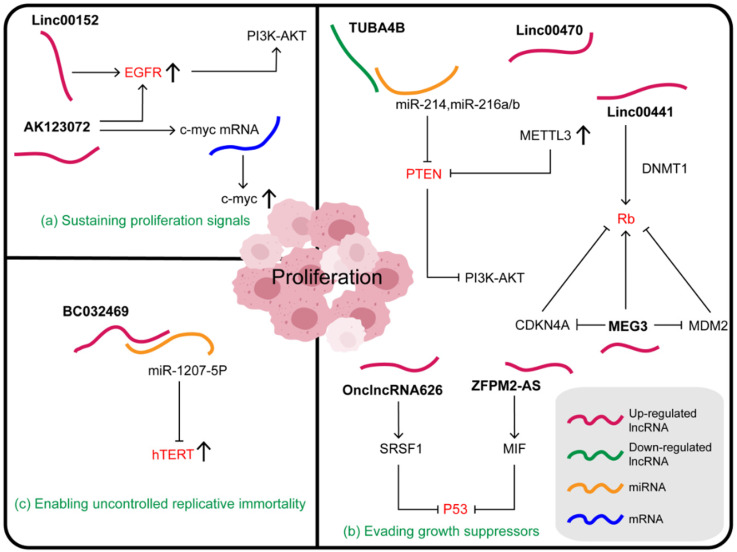
**Relationships between lncRNAs and proliferation in gastric cancer. (a)** Some lncRNAs mainly regulate the ability of tumors to grow by regulating growth factors or receptors. **(b)** Some lncRNAs affect the growth and proliferation of gastric cancer cells by directly or indirectly altering the expression of tumor suppressor factors. **(c)** Some lncRNAs play a role in the proliferation of gastric cancer cells by affecting telomerase activity.

Sustained proliferation is a typical characteristic of cancer cells. Complex molecular pathways and dynamic crosstalk between tumor cells and their microenvironment maintain these characteristics. The ability of a cancer cell to sustain chronic proliferation is its most fundamental characteristic, achieved by the maintenance of growth signals via the autocrine and paracrine growth factor pathways 97. Normal tissues strictly control the production and release of growth-promoting signals, which in turn control cell growth and division to ensure homeostasis and thus maintain normal structure and function 96. Cancer cells regulate these signals to mediate tumor growth, and lncRNAs participate in this process by regulating the growth factors or receptors (**Figure 2A**).

Epidermal growth factor receptor (EGFR) belongs to the family of receptor tyrosine kinases (RTK), commonly over-expressed in tumors and considered as a driver and regulator of tumorigenesis 98. Linc00152 in the cytoplasm can bind directly to EGFR, resulting in increased EGFR expression and activation of the EGFR signaling pathway. In addition, the inhibition of p-EGFR, p-AKT, and p-PI3K (phosphoinositide 3-kinase) in cells treated with Linc00152 shRNA suggested that Linc00152 might induce the constitutive activation of EGFR signaling and activate the downstream PI3K/AKT signaling pathway 99. AK123072 is an intron antisense lncRNA that is frequently upregulated by hypoxia in GC. In hypoxia-induced AK123072 over-expression in GC cells, the CPG island methylation of the EGFR gene is significantly decreased, leading to its increased expression, thereby promoting the spread of cancer cells. Moreover, AK123072 can promote GC migration and invasion by increasing the stability and expression of c-Myc mRNA 100.

In addition to their remarkable ability to induce and maintain positive growth stimulating signals, cancer cells must also avoid the powerful mechanisms that negatively regulate cell proliferation. Several tumor suppressors, such as the classic p53, Rb, and PTEN 101,102, have been found to regulate the cell cycle and inhibit cell growth and proliferation in different ways. Characteristically, they are inactivated in cancer cells. Some lncRNAs affect the growth and proliferation of GC cells by directly or indirectly altering the expression of tumor suppressor factors (**Figure 2B**), for example, p53, a key gatekeeper of the cell. P53 is a stress-induced transcription factor, which can promote cell cycle arrest, apoptosis, and senescence 103. The oncogene ZFPM2-AS1, which is an important upstream regulator of the MIF/P53 axis, promotes the proliferation and inhibits apoptosis of GC cells. The mechanism of ZFPM2-AS1 action involves regulating the expression and subcellular localization of p53 to inactivate the p53 signaling pathway through its physical interaction with MIF at the translational or post-translational level 104. Previous studies have shown that MIF, a tumor promoter, decreases the stability of the p53 protein and prevents its nuclear translocation by physically associating with it 105. Furthermore, it was found that the accumulation of MIF protein induced by ZFPM2-AS1 in GC cells was not due to increased protein synthesis, but was due to increased protein stability 104. OnclncRNA-626 located on chromosome 11 can also affect p53 expression. It interacts specifically with serine- and arginine-rich splicing factor 1 (SRSF1), increasing its stability and upregulating it in GC tissues. RSF1, also known as SF2/ASF, which plays a regulatory role in many cancers, is a member of the SR family. It has two domains, RRM1 and RRM2, where RRM2 domain is the dominant binding site of OnclncRNA-626 106,107. Thus, OnclncRNA-626 may exert its biological function through the SRSF1/p53 pathway and may be a potential therapeutic target in GC 107.

Some lncRNAs also act on the tumor suppressor PTEN, whose frequent deletion in cancer suggests its physiological importance. PTEN governs a variety of biological processes by inhibiting the PI3K/AKT-mammalian target of rapamycin (mTOR) pathway through its lipid phosphatase activity; this includes maintaining genomic stability, cell survival, migration, proliferation, and metabolism 108. TUBA4B, a ceRNA, was found to physically bind to and chelate miR-214 and miR-216a/b, thus increasing the expression of the common downstream target PTEN, decreasing the expression of p-PI3K and p-AKT, and thereby leading to the inactivation of the PI3K/AKT signaling pathway. Moreover, the weakened cell malignancy phenotype induced by TUBA4B was evidently rescued by PTEN silencing and AKT activation 109. LINC00470, mainly distributed in the cytoplasm, can bind to PTEN mRNA and promote its degradation. This process, mediated by N6-methyladenosine (m^6^A) writer METTL3, enhanced its m^6^A methylation level resulting in high levels of m^6^A RNA and METTL3 in GC. It was also found that the LINC00470-METTL3-mediated degradation of PTEN mRNA was dependent on the human YTH domain family 2 (YTHDF2), an m^6^A reader protein 110. LncRNAs that influence m^6^A modification play a key role in regulating cancer progression. For example, lncRNA GAS5-AS1 inhibits the growth of cervical cancer by interacting with RNA demethylase ALKBH5 to increase GAS5 stability and decrease GAS5 m^6^A modification 111. It has also been reported that LINC00470 binds to fused in sarcoma (FUS) and AKT, which anchors FUS in the cytoplasm and activates AKT. Subsequently, the expression of ELFN2 is upregulated, thus affecting glycolysis, inhibiting autophagy, and promoting tumorigenesis 112,113. Moreover, the mechanism of LINC00470 action on tumor suppressor PTEN also supports its influence on gastric cancer.

Finally, the key tumor suppressor Rb can also be regulated by lncRNAs. The Rb protein integrates signals from different extracellular and intracellular sources and determines whether the cell should continue its growth and division cycle 101. When cells are stimulated by growth-promoting signals, cyclin D/CDK4 is induced to phosphorylate Rb1, so that the Rb-E2Fs complex releases E2Fs and cells can enter S-phase 114. Linc00441, a carcinogenic lncRNA, recruits DNMT1 into the Rb1 promoter and inhibits the expression of RB1 in GC cells, leading to their proliferation 77. In addition to the previously mentioned effects on the expression of ATG4B, MEG3 can inhibit MDM2, increase the active form of Rb, and then enhance the interaction between Rb and transcription factor E2Fs to block the transcription of its target gene. Additionally, MEG3 influences Rb via the regulation of RNA-protein binding or CDKN4A 115.

Telomerase, a DNA polymerase that adds telomeric repeats to the ends of telomeric DNA, maintains telomere length and protects the chromosome ends, crucial for the development and survival of tumors 96,116. Telomerase reverse transcriptase (hTERT), a catalytic subunit of telomerase, plays an important role in maintaining telomere length, maintaining cell division, and replication 117. A novel endogenous lncRNA BC032469, which is highly expressed in GC tissue, can combine directly with, and effectively act as a sponge for, miR-1207-5p to regulate hTERT expression and thus the cell cycle in the G0/G1 phase. Therefore, upregulation of BC032469 is associated with larger tumor volume, poor differentiation, and shorter survival time in patients with GC (**Figure 2C**) 90.

### LncRNAs and resistance to cell death

**Figure 3:**
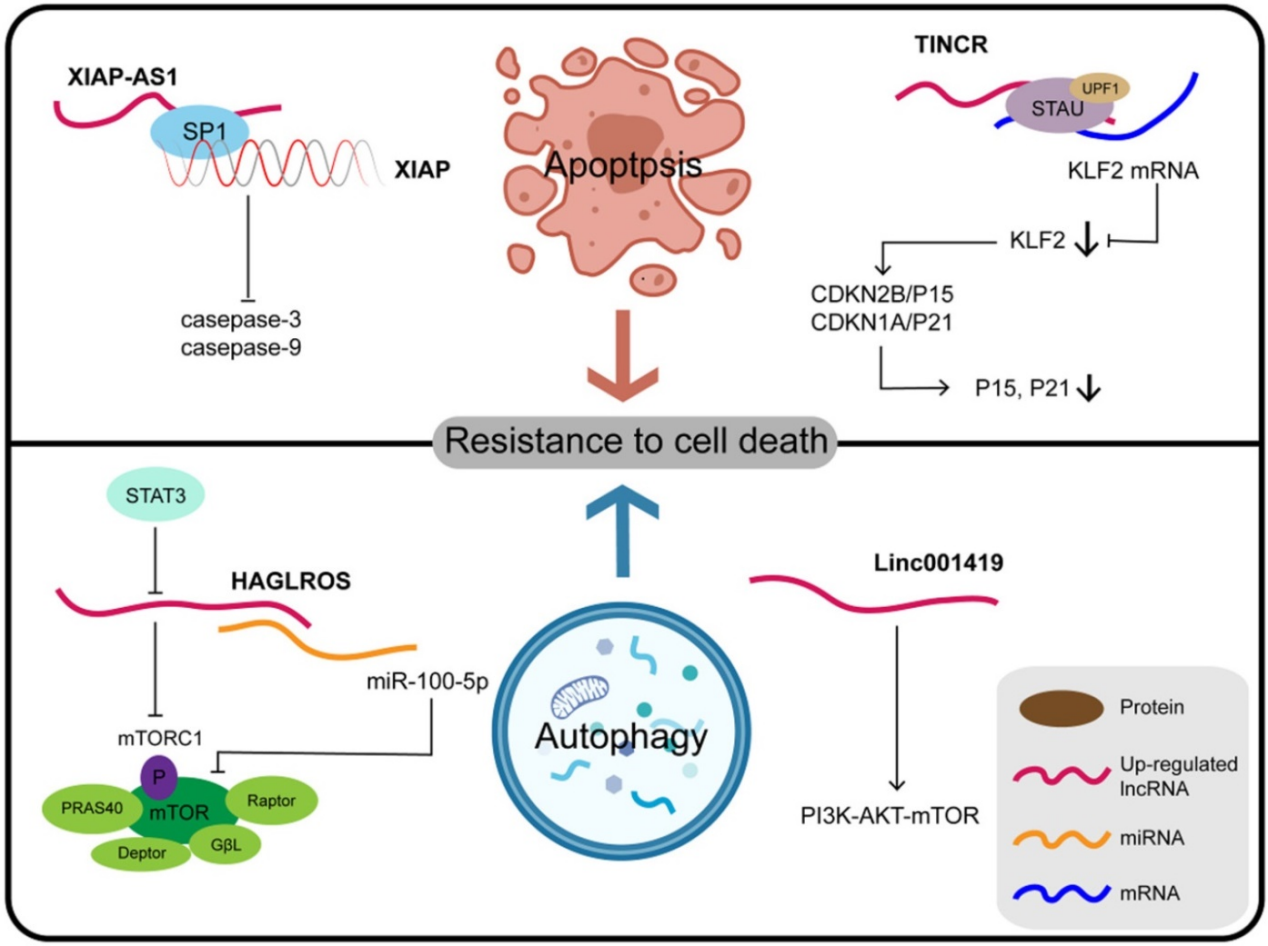
Some lncRNAs and their molecular partners or genomic targets play their key roles in resistance to cell death in gastric cancer.

Programmed cell death (PCD) refers to apoptosis, autophagy, and programmed necrosis, all of which are mediated by intracellular programming. The fate of tumor cells can be determined by the balance between cell death and survival mediated by PCD. Abnormal PCD processes can also result in faulty proliferation patterns of tumor cells. The cascade of events due to lncRNAs involved in tumorigenesis and malignant transformation are mostly related to the first two forms, namely apoptosis and autophagy (**Figure 3**). Another newly-described lncRNA, XIAP-AS1, mainly located in the nucleus, is transcribed from the first intron of the complementary chain of the XIAP (X-linked apoptosis inhibitor) gene. XIAP binds to the BIR2 and BIR3 domains of caspase-3 and caspase-9, respectively, to inhibit their activation and block apoptosis. Full-length XIAP-AS1 RNA interacts with SP1, which participates in the transcription of XIAP, and thus affects the apoptosis process mediated by XIAP. Furthermore, the downregulation of XIAP-AS1 promotes the apoptosis of gastric tumor cells induced by tumor necrosis factor (TNF)-related apoptosis-inducing ligand (TRAIL) 118,119. TRAIL, a member of the TNF family of cytokines, induces apoptosis through both mitochondrial-dependent and death receptor pathways in tumor cells, preferentially killing tumor cells but not normal cells 120. Therefore, XIAP-AS1 affects the apoptosis of GC cells by influencing the transcription of XIAP and acting as a potential target for TRAIL. Silencing the expression of TINCR inhibits cell proliferation, colony formation, tumorigenicity, and can promote cell apoptosis as reported for SGC7901 and BGC823 cell lines. TINCR binds to STAU1 protein, which can induce mRNA degradation, and the resulting complex affects the expression of Kruppel-like factor (KLF)2 through this STAU1-mediated mRNA degradation (SMD) 121. SMD is a translation-dependent mechanism that occurs when STAU1, together with the nonsense-mediated mRNA decay factor UPF1, binds at a location downstream of the termination codon 122. SMD is a translation-dependent mechanism that occurs when STAU1, together with the nonsense-mediated mRNA decay factor UPF1, binds at a location downstream of the termination codon 123. When the expression of KLF2 is regulated by TINCR, KLF2 further regulates the transcription and expression of the CDK genes CDKN1A/P21 and CDKN2B/P15, thus affecting the proliferation and apoptosis of GC cells 121. Further understanding of the function and clinical significance of TINCR and its target genes KLF2, CDKN1A/P21, and CDKN2B/P15 may be helpful for early diagnosis and treatment of GC. LINC01419 silencing inhibits the activation of the PI3K/AKT1/mTOR pathway, as demonstrated by reduced phosphorylation of AKT1 and mTOR, thus promoting autophagy of GC cells as well as inhibiting tumor growth and metastasis. These findings offer a new perspective in that the downstream PI3K/AKT1/MTOR axis mediated by LINC01419 may be a new target for GC therapy 124.

### LncRNAs and metastasis

**Figure 4:**
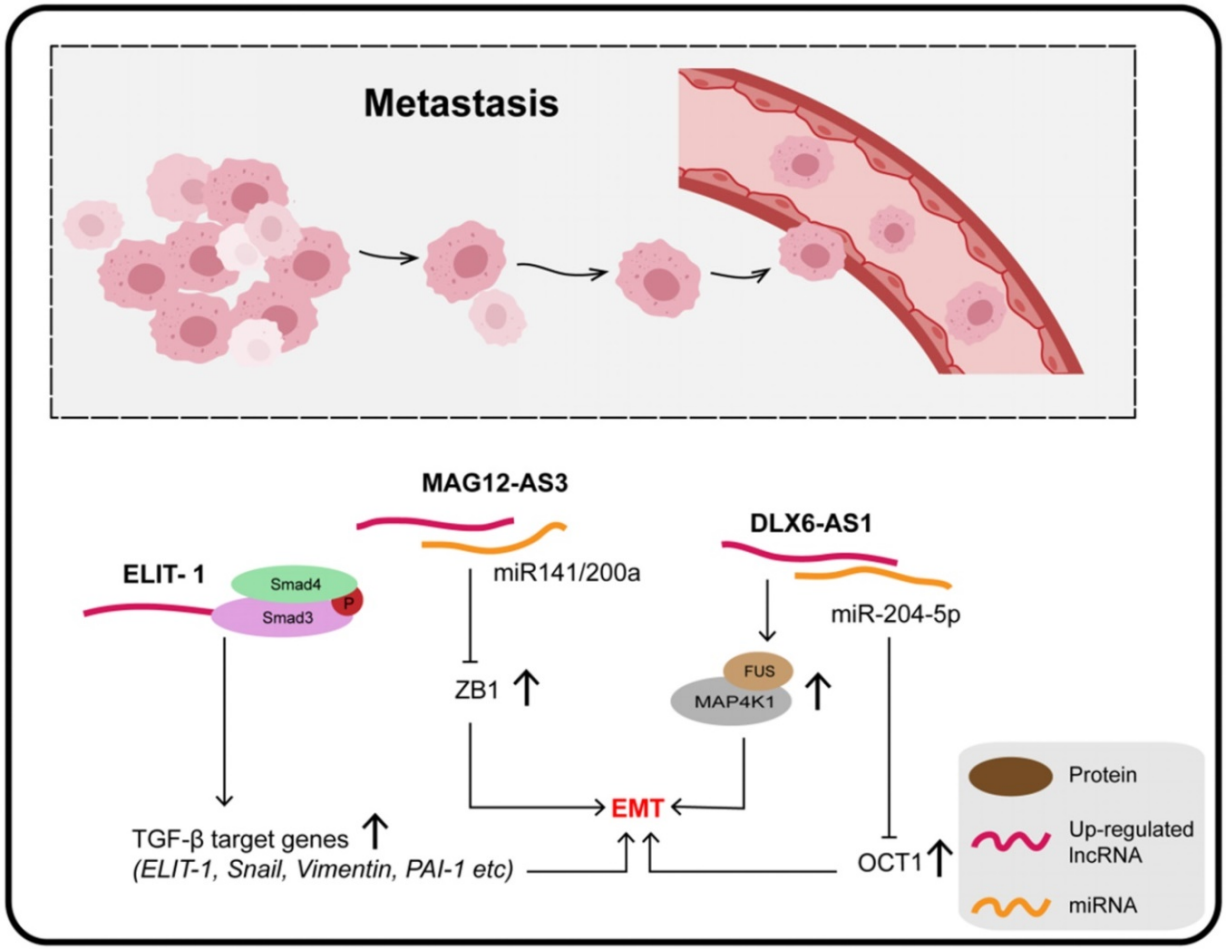
Some lncRNAs play their important roles in the metastasis and invasion of gastric cancer by participating in the EMT process.

EMT, considered to be an important mechanism of tumor metastasis, is a cell program that is crucial for embryogenesis and wound healing, but also furthers malignant progression and can facilitate tumor initiation and metastatic potential of cancer cells. The process of EMT encompasses the disintegration of intercellular connections, reorganization of the actin cytoskeleton, and enhancement of cell viability and invasive capacity 125. The destruction of tight junctions is related to the loss of the epithelial marker E-cadherin and the acquisition of mesenchymal markers N-cadherin and vimentin 126. A variety of lncRNAs is involved in the development of GC by regulating EMT and metastasis (**Figure 4**).

Four molecular subtypes of GC can be defined on the basis of microsatellite stability (MSS) or instability (MSI) and p53 status, namely, MSS/TP53^+^, MSS/TP53^-^, MSI, and MSI/EMT 127. Zhang et al. quantified lncRNA MAGI2-AS3 in these four subtypes of GC and reported that it was extremely high in the MSS/EMT subtype, suggesting that MAGI2-AS3 is associated with EMT. Moreover, miR-141 and miR-200a are known to be negative regulators of the ZEB1 gene and EMT pathway; MAGI2-AS3, mainly located in the cytoplasm, promotes tumor metastasis by binding miR-141/200a to maintain downstream ZEB1 over-expression, which is a well-accepted core factor regulating the EMT process 128,129.

It was confirmed that knockout of DLX6-AS1 inhibits the expression of N-cadherin, MMP9, and SLUG, as well as promoting the expression of E-cadherin. In addition, the distribution of the actin cytoskeleton and the presence of filamentous pseudopodia were significantly reduced when DLX6-AS1 was knocked out. The specific mechanism of its action involves DLX6-AS1 functioning as an endogenous cancer-promoting ceRNA that upregulates OCT1 by binding with miR-204-5p, which is downregulated in GC and inhibits tumor progression through various mechanisms. The 3-UTR region of OCT1 contains a potential binding site for miR-204-5p, and has been found to be a transcription factor contributing to carcinogenesis in GC and precancerous lesions. In addition, OCT1 was confirmed to enhance the expression of DLX6-AS1 by targeting the promoter region, revealing a positive feedback loop (OCT1/DLX6-AS1/miR-204-5p/OCT1) which promotes the progress of GC and EMT 130. Together, DLX6-AS1 is upregulated in GC tissues and cell lines and is associated with distant metastasis, T3/T4 invasion, and poor clinical prognosis.

### LncRNAs and DNA damage

**Figure 5:**
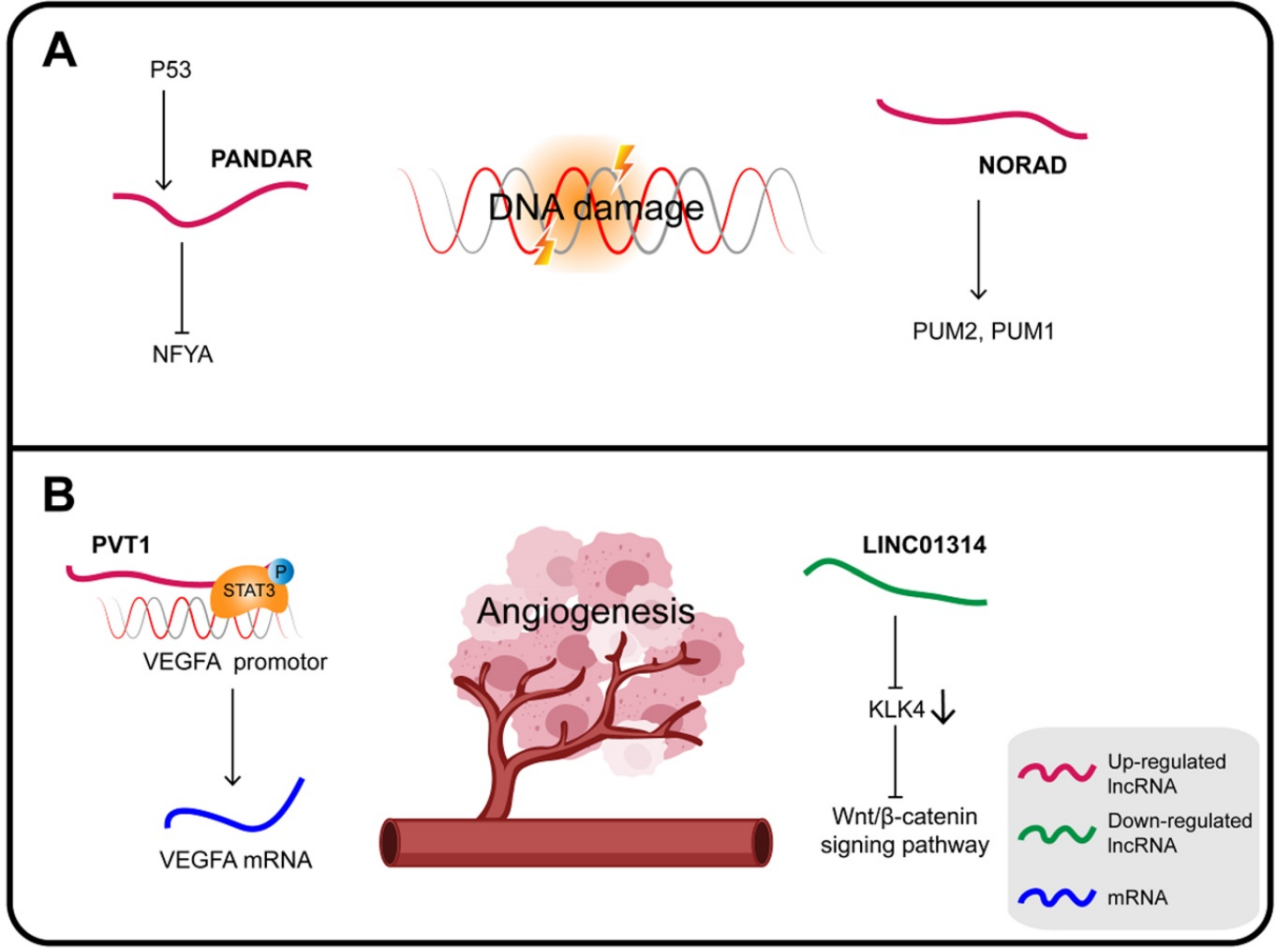
** (A)** DNA damage involves a complex regulatory network and some lncRNAs can also participate in the regulation of DDR to affect cancer progression in gastric cancer. **(B)** Some lncRNAs have been shown to play a role in the development of gastric cancer by regulating angiogenesis.

An underlying feature of cancers is genomic instability, which is associated with a greater tendency to trigger DNA damage responses (DDR) 131. The DDR which involves a complex regulatory network of lncRNAs may also participate in cancer progression (**Figure 5A**). For example, the NF-κB-HOTAIR axis links DDR to ovarian carcinogenesis 132, and LINC00261, an epigenetically regulated tumor suppressor, is critical for the activation of DDR in lung cancer 133.

In GC, PANDAR (promoter of CDKN1A antisense DNA damage-activated RNA), whose expression is induced by p53, is also a direct transcriptional target of the p53 protein. PANDAR is induced in response to DNA damage via inhibition of the function of the transcription factor nuclear transcription factor Y subunit alpha (NFYA). Upregulated HOTAIR inhibits the expression of p53 by enhancing the p53 promoter H3K27me3, while overexpression of PANDAR increases the stability of p53 in response to DNA damage 134.

Noncoding RNA activated by DNA damage (NORAD), a highly conserved lncRNA essential for mitotic cell division, plays a key role in DNA replication and DDR. NORAD responds to DNA damage by regulating the activity of the RNA-binding proteins PUM2 and PUM1 in order to maintain genomic integrity 135. NORAD expression is upregulated in GC, which can also significantly increase FOXO6 abundance and promote tumor progression. Mechanistically, NORAD acts as a ceRNA sponge for miR-608 to upregulate the expression of FOXO6 that in turn enhances the tumorigenicity of GC cells by upregulating the oncogene c-Myc 136.

### LncRNAs and angiogenesis

Angiogenesis is an essential feature of cancer, providing nutrients and oxygen for tumor growth. VEGFA, a major regulator of angiogenesis, can induce the proliferation and migration of endothelial cells to form new blood vessels 137 (**Figure 5B**).

PVT1 is considered an oncogene interacting with different proteins and playing a variety of roles in carcinogenesis. In GC, PVT1 promotes cell proliferation by binding to EZH2 and also promotes migration and invasion by interacting with FOXM1 138. In addition, PVT1 can bind to nuclear p-STAT3 to form a complex that protects p-STAT3 from degradation in the polyubiquitin-proteasome, and then activates the STAT3/VEGFA axis to induce angiogenesis. Activated STAT3 in turn triggers PVT1 transcription, maintaining the oncogenicity of the PVT1/STAT3/VEGFA axis 139. Therefore, the upregulation of lncRNA PVT1 is closely related to the high density of microvessels in GC and its poor prognosis; it can promote the proliferation of GC cells *in vitro* and tumor growth and angiogenesis *in vivo*.

The KLK4 gene is a direct target of LINC01314 that is poorly expressed in GC. KLK4 is a subunit of a serine endopeptidase found in a variety of cells and tissues, which is essential for diversified pathophysiological development, including angiogenesis in cancers. Over-expression of LINC01314 or KLK4 silencing suppresses angiogenesis in GC cells through negative regulation of the Wnt/β-catenin signaling pathway. At the same time, the tumor weight, the microvessel density of the transplanted tumor and, most importantly, the expression of both VEGF-C and VEGFR-3 were all decreased. In addition, LINC01314 over-expression can decrease N-cadherin and increase E-cadherin expression, therefore presumably it can also inhibit GC migration 140,141.

### LncRNAs and the immune response

**Figure 6:**
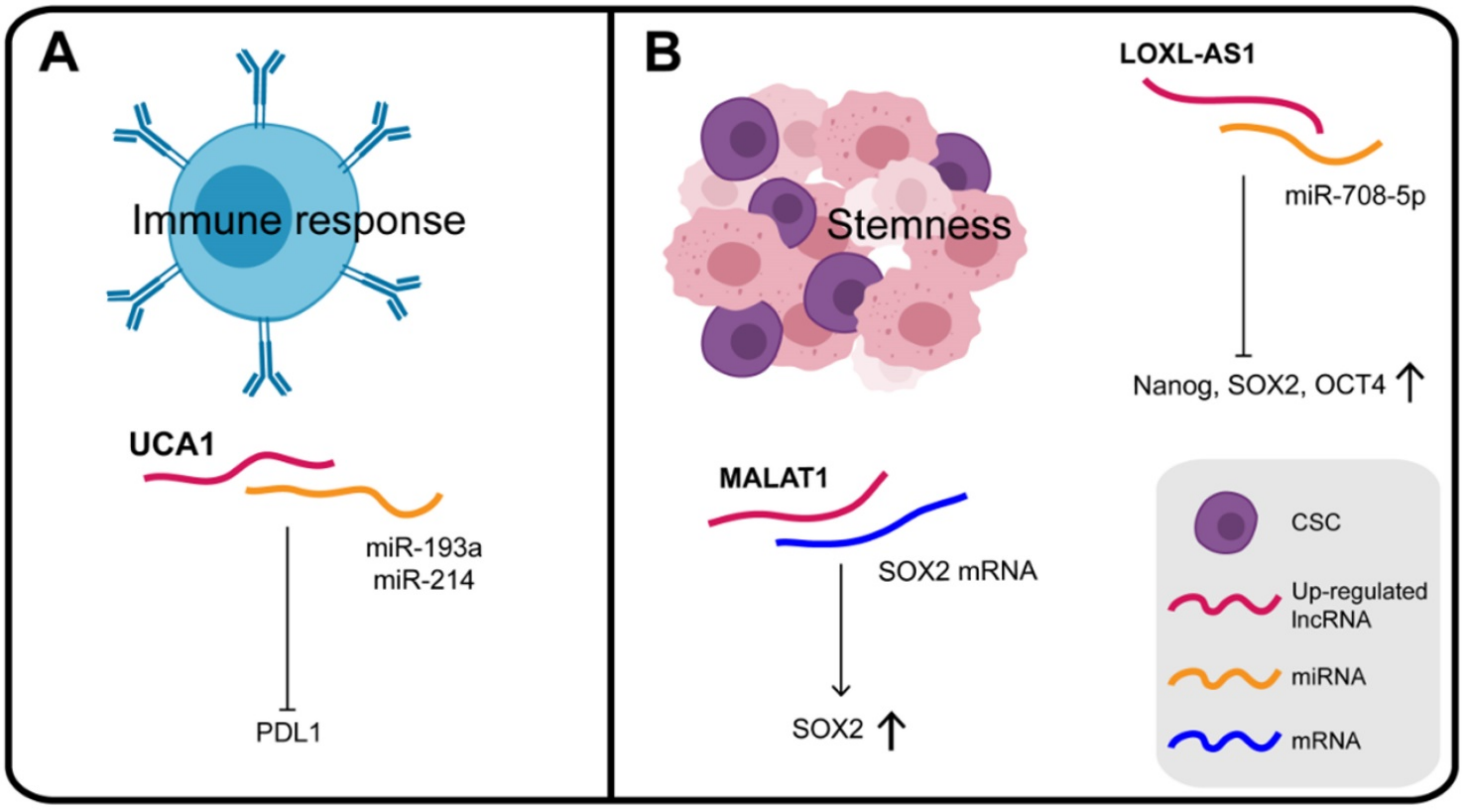
** (A)** Some lncRNAs play the regulatory roles in the immune system and participate in the balance between the immune system and gastric cancer cells. **(B)** Some lncRNAs play the key roles in maintaining the gastric cancer stem cell phenotype by influencing their expression.

The differentiation and activation of innate and adaptive immune cells is highly dependent on a coordinated set of transcriptional and post-transcriptional events. LncRNAs are now emerging as important regulators of the differentiation and activation of immune cells. They do not directly encode innate or adaptive immune proteins but regulate the differentiation and function of immune cells, such as dendritic cell activity, T cell ratios and metabolism 142. A dynamic equilibrium exists between the immune system and the malignant tumor, which can evade immune clearance and proliferate pathologically. Studies are increasingly revealing that lncRNAs have regulatory roles in the immune system, providing a reliable research basis for lncRNAs to participate in the immunotherapy of GC (**Figure 6A**). For instance, in addition to the effects of UCA1 on chromatin in GC, it can also act as a "miRNA sponge" for miR-193a and miR-214, reducing their targeting of the 3′ UTR of PDL1 and thus facilitating PDL1 expression 80. PDL1 is a transmembrane protein, overexpressed in cancer cells, which suppresses the host's immune system by binding to PD1 on activated T cells, B cells, and bone marrow cells 143. Therefore, UCA1, which protects PDL1 from the inhibitory effect of miRNAs and promotes immune escape of GC cells, may be a potential target, providing a new direction for immunotherapy of GC.

In general, based on the theory of their binding with miRNAs and proteins, many types of lncRNAs found in exosomes could participate in the process of tumorigenesis as tumor antigens to activate anti-tumor immune response, and may thus be considered as targets for immunotherapy. However, the regulatory mechanism of existing RBPs such as lncRNA-miRNA-mRNA on immune regulation of GC is still unclear and requires further exploration.

### LncRNAs and the stemness of gastric cancer

CSCs (cancer stem cells), recognized as tumor-initiating cells, are tumor cells with the capacity for self-renewal, metastasis, tumorigenicity, which exhibit chemotherapy resistance 144 (**Figure 6B**). Numerous studies have confirmed the genetic homology of lncRNA LOXL1 antisense RNA 1 (LOXL1-AS1) in various human cancers. Thus, it was confirmed that LOXL1-AS1 knockdown decreased the expression of stem cell factors such as Nanog, SOX2, and OCT4. Independently, MKN45 cells with lower expression of LOXL1-AS1 were found to be more sensitive to cisplatin therapy, which further supported the notion that LOXL1-AS1 promotes the maintenance of CSC features in GC 145. In GC cells over-expressing SPRY4-IT1, CSC markers, including CD73, CD146, and ALDH1, were all significantly elevated. In contrast, inhibition of SPRY4-IT1 reduced the self-renewal ability of BGC823 cells and significantly reduced the expression of CSC markers 146. These results consistently show that SPRY4-IT1 plays a role in maintaining the GC stem cell phenotype.

## Potential clinical applications of lncRNAs in gastric cancer

### LncRNAs as potential novel biomarkers

**Figure 7:**
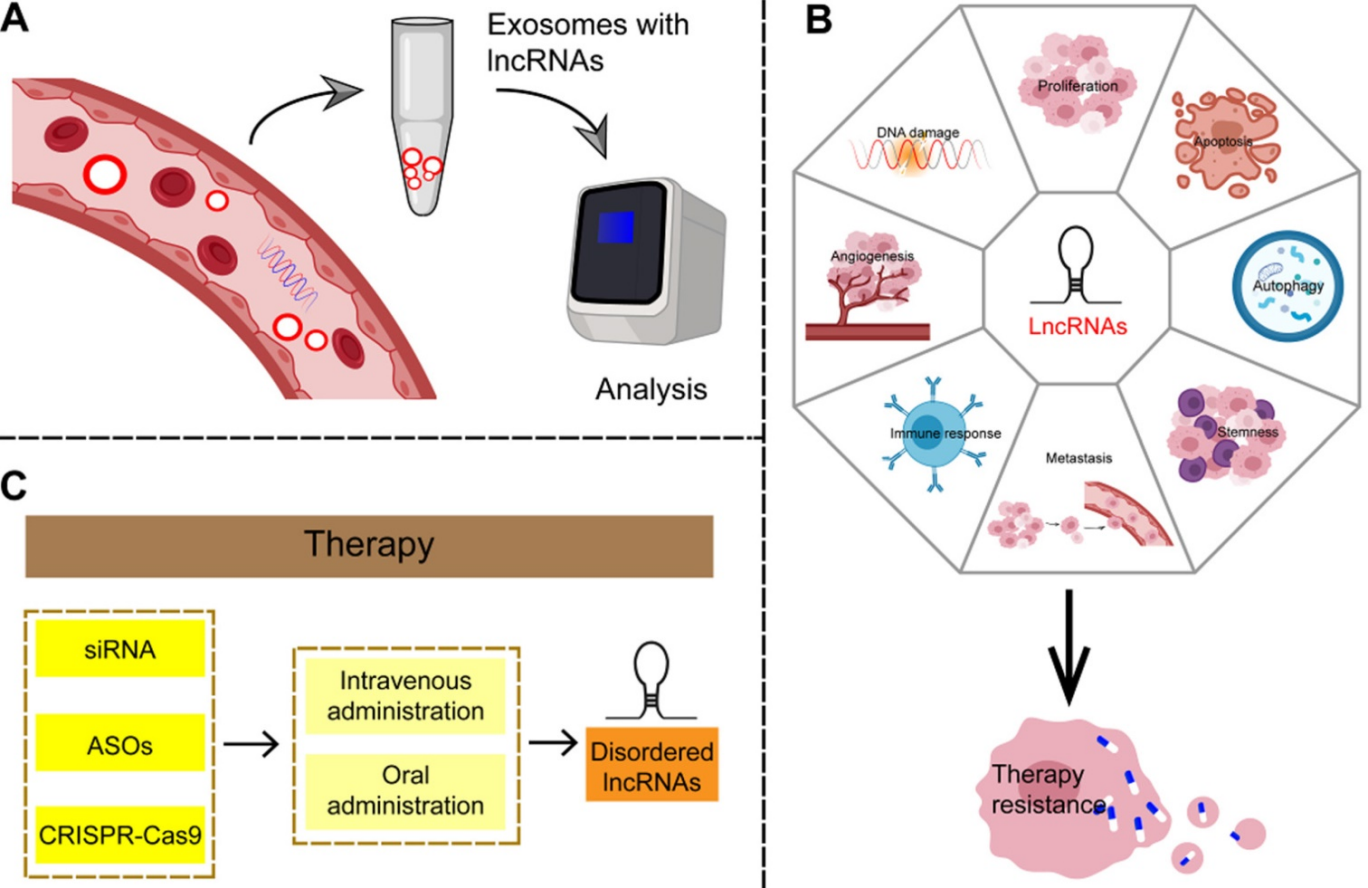
** Potential clinical applications of lncRNAs in gastric cancer. (A)** LncRNAs are enriched and stable in exosomes, can be detected in plasma samples, and are considered as non-invasive and promising biomarkers for gastric cancer.** (B)** LncRNAs can be involved in the complex regulatory network of therapy resistance in a number of ways, including affecting the amplification or overexpression of oncogenes, suppression of tumor suppressor genes, immune escape, apoptosis, autophagy, EMT, angiogenesis, tumor stemness and so on.** (C)** Given the irreplaceable role of lncRNAs in the development of gastric cancer, targeting lncRNAs may produce reliable therapeutic results.

Because the majority of GC patients is diagnosed in the unsalvageable late stage, there is an urgent need to identify effective biomarkers for early disease. Some established circulating tumor-specific biomarkers, such as carcinoembryonic antigen (CEA), carbohydrate antigen (CA)242, CA724, and serum pepsinogen (SPG), are known to be of limited use in the diagnosis of GC 165. LncRNAs can be detected in a highly stable form in plasma and are therefore considered to be non-invasive and promising biomarkers for cancer diagnosis (**Figure 7A and Table 3**). Studies have shown that cancer cells secrete exosomes, and lncRNAs are enriched and stabilized within these 166. Exosomes, small vesicles with a diameter of about 30-150 nm that act as mediators of cell-to-cell communication, play an important role in the process of tumor proliferation and metastasis by delivering carcinogens 167. As carriers of information between cells, exosomes represent a hitherto unknown biological communication system. However, the biological function of secretory lncRNAs in GC remains to be elucidated. LncRNAs such as ZFAS1, HOTTIP, and lncUEGC1 can be detected in plasma exosomes; significant differences between cancer patients and healthy controls were observed in their expression and area under the curve (AUC) values by receiver operating characteristic curve (ROC) analysis 168-170. Therefore, a large number of lncRNAs has been found to be suitable as noninvasive biomarker candidates for GC.

Evidence indicates that plasma H19 levels can be used to distinguish patients with early GC from controls, with clinical results that are as satisfactory as traditional tumor biomarkers. After further analysis of 70 patients and 70 controls, it was concluded that the level of plasma H19 in patients with GC was significantly higher than in healthy controls (p < 0.0001); ROC analysis revealed that the AUC was 0.838 (p < 0.001; sensitivity, 82.9%; specificity, 72.9%). Furthermore, the expression of H19 distinguished early-stage GC from controls with an AUC of 0.877 (sensitivity, 85.5%; specificity, 80.1%). It has also been demonstrated that plasma H19 levels are stable during direct treatment with RNase and are therefore stable and detectable in plasma 171. Therefore, plasma H19 may be a promising tumor marker for GC; however, many questions remain to be resolved before these findings can be translated into clinically useful, non-invasive screening strategies for GC patients.

In addition to the use of a single lncRNA in the diagnosis of GC, studies have shown that multiple plasma lncRNA combinations can provide more effective diagnostic power for GC diagnosis. Genome-wide plasma lncRNA profiles were screened to identify promising GC lncRNA biomarkers, with the result that plasma levels of FAM49B-AS, GUSBP11, and CTDHUT in GC patients were found to be significantly higher than in healthy controls after validation with data from 446 subjects (AUC of 0.818, 95% CI, 0.772-0.864). Moreover, the combination of these three lncRNAs with CA242 or CA724 was superior to the traditional CA242 or CA724 biomarkers alone for GC diagnosis; however, the AUC value of the combination (0.784) was lower than for the three lncRNAs. These three lncRNAs were stable and detectable in human plasma, and their amounts decreased significantly on the 10th day after surgical resection 172. Therefore, the plasma lncRNAs FAM49B-AS, GUSBP11, and CTDHUT have great potential as non-invasive biomarkers for the diagnosis of GC.

Evaluation of prognosis post-treatment is also essential for cancer patients. It can be used to assess the treatment status and provide evidence for adjusting treatment regimens. In recent years, together with tumor diagnostic biomarkers, the study of prognostic biomarkers related to lncRNAs has also made great progress (**Table 4**).

CASC19, also known as CARLo-6 or LINC01245, an intergenic lncRNA 324 bp in length, is encoded on chromosome 8q24.21. An innovative genomic analytical method has combined different lncRNA expression profiles with WGCNA, and resulting in the definition of CASC19 as a key lncRNA related to the development and progression of AGC. CASC19 was found to be upregulated in AGC tissue, and to correlate positively with abnormal clinicopathologic parameters. These included pathologic T stage, TNM stage, lymph node metastasis, and poorer prognosis in patients with AGC. Overall survival of patients with high CASC19 expression was significantly worse than patients with low CASC19 expression. In this cohort of 375 patients, CASC19 was therefore suggested to be an independent prognostic factor for AGC overall survival 173. Thus, CASC19 is involved in the development of AGC through the regulation of MAPK, calcium, Wnt, and insulin signaling pathways and may be a new prognostic marker and potential therapeutic target for the disease.

The degree of over-expression of lncRNA NEAT1 in 140 GC specimens was found to be positively correlated with clinical stage, histological type, and distant or lymph node metastasis. Patients with low levels of NEAT1 had higher survival rates than those with high levels. Both univariate and multivariate analysis showed that overexpression of NEAT1 was an independent factor of poor prognosis in patients with GC. Knockout of NEAT1 significantly inhibited migration and invasion of GC cells *in vitro* and regulated the expression of EMT-related proteins. NEAT1 has also been shown to play a role in tumor development in esophageal cancer and glioma cells 174. Thus, NEAT1 participates in the occurrence and development of GC and can be employed as a biomarker for its prognosis.

### LncRNAs and resistance to therapy

Chemoradiotherapy (CRT) is the primary clinical treatment for multiple cancers including GC. Nevertheless, the development of acquired resistance to CRT greatly reduces its efficacy. Thus, resistance is the key mechanism of cancer escape from therapeutic attack and also the main obstacle for tumor therapy. Therefore, urgent exploration of the mechanisms of CRT resistance is required in order to improve therapeutic effects and reduce adverse reactions. Recent evidence suggests that lncRNAs may play vital roles in GC resistance to CRT (**Figure 7B and Table 5**).

LncRNA CRAL can regulate CYLD expression by binding to miR-505 and acting as an endogenous sponge, which can then reverse cisplatin resistance by increasing DNA damage and enhancing apoptosis. CYLD, a de-ubiquitinase, is considered a negative regulator of the PI3K/AKT/NF-κB signaling pathway. It was shown that in CRAL-deficient GC cells, CYLD expression was decreased and the activation of AKT increased. Blocking the PI3K/AKT signaling pathway reversed cisplatin resistance induced by CRAL deficiency and rendered GC cells sensitive to chemotherapeutic drugs. Thus, the suitability of AKT inhibitors for patients with GC could be determined based on the CRAL expression level. In summary, lncRNA CRAL may be a potential biomarker and therapeutic target for overcoming cisplatin resistance in GC by acting as a ceRNA via the miR-505/CYLD/AKT axis 188.

The autophagic adapter SQSTM1 (also known as the autophagic receptor) which recruits polyubiquitinated proteins to LC3-positive autophagosomes for degradation, can also recruit ARHGAP5-AS1 for autophagic degradation. However, in drug-resistant cancer cells with impaired autophagy, the amount of ARHGAP5-AS1 is upregulated and drug resistance increased. RBPs play an important role in regulating the stability of mRNA; it was also found that m^6^A modification can affect the stability of mRNA through RBPs such as HuR. ARHGAP5-AS1 modified ARHGAP5 mRNA by recruiting METTL3 for m^6^A modification and HuR binding, stimulated the transcription of ARHGAP5 in the nucleus, and also stabilized ARHGAP5 mRNA in the cytoplasm 189. Therefore, targeting the ARHGAP5-AS1/ARHGAP5 axis may be a promising strategy for overcoming chemoresistance in GC.

Abnormal expression of PCAT1, an important regulator of multidrug resistance, is found in many cancers. For example, upregulation of PCAT1 in esophageal cancer tissue reduces the sensitivity of cancer cells to cisplatin *in vitro* 190. Functional studies on Caco-2 and HT-29 cells showed that silencing PCAT1 increased the sensitivity of the cells to 5-fluorouracil by upregulating the expression of c-Myc 191. PCAT1 was also significantly upregulated in CDDP-resistant GC tissues and cells, and caused cisplatin resistance in GC cells by binding to EZH2 to increase H3K27me3, that silenced PTEN 150. In conclusion, PCAT1 is a positive regulator of cisplatin resistance in cancer cells and targeting PCAT1 may be an effective drug resistance strategy.

To sum up, a variety of lncRNAs has been implicated as regulators of downstream gene expression and/or function via their action as ceRNAs or via directly acting on proteins, thereby regulating drug resistance. They can participate in the complex regulatory network of CRT by affecting the amplification or overexpression of oncogenes, suppression of tumor suppressor genes, immune escape, apoptosis, autophagy, EMT, programmed death, tumor stemness and potentially by other mechanisms as well. Targeting these maladjusted lncRNAs may be a promising strategy to reverse CRT-induced resistance. In addition, targeted treatment of GC with lncRNAs in combination with traditional chemotherapeutic agents may be a promising alternative to reversing drug resistance and may help improve the prognosis of patients with AGC. This approach may ultimately open up new potential avenues for overcoming therapy resistance.

### Therapeutic strategies targeting specific lncRNAs

Regulatory lncRNA networks can mediate anti-proliferative effects and reduce invasion of GC, thus mediating therapeutic effects, and making it possible to consider exploiting lncRNAs as therapeutic targets in GC. One advantage of targeting lncRNAs is that functional ablation of a lncRNA with regulatory function may have pleiotropic effects, resulting in simultaneous damage to multiple tumor-related pathways. Therefore, targeting lncRNAs may yield reliable therapeutic results that reduce drug resistance in tumors. Small interference RNAs (siRNAs) targeting abnormal GC-specific lncRNAs may be an effective strategy in this respect 196. Antisense oligonucleotides (ASOs) can also correct aberrant lncRNA networks by inducing RNase H-dependent degradation or by spatially blocking lncRNA activity. In addition, specific chemical modification of lncRNA-targeted ASOs enhances their effectiveness and stability and allows free absorption *in vivo* without the need for transfer methods 197. In addition, the regulation of lncRNA expression based on CRISPR-Cas9 has been demonstrated as a therapeutic tool for clinical cancer therapy 198. Although there is no clear evidence that this approach can be useful in GC, it is worth further exploring this avenue based on these theories. Finally, because lncRNAs are emerging new targets but with an as-yet incomplete understanding of their mechanisms of action, it is important to use controls in gene silencing studies to ensure that the observed phenotypes are caused by the interaction of the targets. In addition, some challenges, such as missed target effects, modes of targeted delivery, and activation of the immune response, need to be resolved before these lncRNAs can be considered for clinical application 199. Although most studies to date on the use of lncRNA-targeted drugs for GC are still in the preclinical phase, they nevertheless highlight the potential of this strategy (**Figure 7C**).

## Conclusions and perspectives

In view of the high mortality and typically delayed diagnosis of gastric cancer (GC), a comprehensive understanding of the molecular pathogenesis and potential biomarkers of this disease is urgently needed to supplement existing treatment options. With the development of high throughput sequencing techniques such as microarrays or RNA-seq, important roles of various lncRNAs have been revealed. These have attracted the attention of investigators for their relevance also in the pathogenesis of GC. Large numbers of lncRNAs have been identified as being pertinent for pathology caused by imbalances in gene regulation and the resulting abnormalities in biological processes in GC, mainly due to the circumstantial evidence of their often-abnormal expression in GC. In recent decades, studies have shown that lncRNAs regulate gene expression at transcriptional, post-transcriptional, and epigenetic levels, and participate in the occurrence, development, and progression of GC.

Screening of lncRNAs revealed that most of them have a promoting effect on GC, for example, Linc00152 and PANDA promote proliferation and growth and MAGI2-AS3 promotes tumor invasion and metastasis; however, only a few lncRNAs inhibit GC *in vitro* and *in vivo*, including PWRN1 and TUBA4B that inhibit the proliferation of GC cells. Irrespective of their promoting or inhibiting action on tumor growth, the characteristics of lncRNAs involved in the regulation of GC makes them theoretically potential biomarkers for GC diagnosis, prognosis, prediction and recurrence, some of which have also progressed to clinical trials. For example, PC antigen 3 (PCA3) detection test has been developed to assess prostate cancer risk using urine samples; this indicates progress and provides encouragement for further investigations 200. Compared with traditional biomarkers, lncRNAs have the following advantages: (1) They have higher specificity and sensitivity. (2) LncRNAs testing is a simple, non-invasive, and easy-to-accept method. (3) LncRNAs can be used as biomarkers independently, in combination with other lncRNAs, or as a consummate complement to classic cancer biomarkers. Moreover, because of the tissue- and disease-specific expression patterns of lncRNAs and their ability to control tumor micro-regulatory networks, they are of great significance not only as biomarkers but also as therapeutic targets. Thus, this is a promising field for molecular targeted therapy of GC. Strategies for targeting and regulating lncRNAs based on siRNAs, ASOs, CRISPR-Cas9, etc. are currently being explored, which will contribute to the development of more effective lncRNA-based GC treatment methods.

Moreover, some new types of lncRNAs have good prospects for applications and are worth exploring. Hitherto, some PROMPTs have been found to regulate cancer biology by interacting with adjacent proteins, and may also represent new biomarkers or therapeutic targets. Although there are few reports of PROMPTs specifically in the context of GC, they may provide new directions for future research on GC-related lncRNAs. Similar to PROMPTs, there is little data on eRNAs in GC, which may also gradually become a research hotspot.

Currently, there are numerous challenges for translating knowledge on lncRNAs to the clinic. First, their application must be based on knowledge of their mechanisms of action *in vivo* and *in vitro*, but only some of these have been well probed and elucidated. For example, how some lncRNAs affect DNA hydroxymethylation, chromatin remodeling, or alternative splicing of mRNAs in GC is still being explored. Owing to the complex nature of the tumor micro-environment and the involvement of multiple lncRNAs, we need to identify more lncRNAs affecting GC and construct a network of regulatory interactions between lncRNAs. Moreover, because lncRNAs function primarily through interactions with other biomolecules, elucidation of the secondary structure of their interaction domains is particularly necessary. The analysis of these lncRNA binding motifs may result in the identification of new RNA-based targets for disease prevention and treatment. At present, the study of lncRNAs in GC is mainly focused on their expression profiles, and the role of lncRNA mutants in gastric carcinogenesis needs further study. In addition, the low accessibility of lncRNAs is also a problem. Such problems can be tackled with the help of animal models and a new generation of technologies. The integration of functional lncRNAs and GC biology will further our understanding of the mechanisms involved in GC carcinogenesis. Although the application of specific lncRNAs to diagnosis, prognosis, or treatment of GC is still a distant goal, there is no doubt about their significance. A comprehensive exploration of this still-mysterious realm will be expected to provide more new strategies for the prevention and treatment of GC in the near future.

## Figures and Tables

**Table 1:** Multi-functional roles of lncRNAs in gastric cancer

Mode of mechanism	LncRNA	Expression	Molecular mechanism	Reference
Histone Modification	UCA1	up	Mediates the trimethylation of H3K27 in promoters of p21 and SPRY1 by binding to EZH2.	24
	MNX1-AS1	up	Promotes GC progression through EZH2/BTG2 and miR-6785-5p/BCL2 aixs	29
	SNHG3	up	Regulats neighboring MED18 gene methylation.	30
	ARHGAP27P1	down	Epigenetically regulates p15 and p16	32
	GCLNC1	up	Acts as a modular scaffold of WDR5 and KAT2A complexes	33
	GCAWKR	up	Acts as a molecular scaffold of WDR5/KAT2A complexes	34
	LINC00673	up	Acts as a scaffold for LSD1 and EZH2	35
	FEZF1-AS1	up	Represses the expression of P21 via binding with LSD1	21
	HOTAIR	up	Recruits the PRC2 complex to silence target gene via H3K27me3 modification	71
	XIST	up	Interacts with EZH2 to suppress transcription of its potential target KLF2	72
	TUG1	up	Associates with PRC2	73
	AGAP2-AS1	up	Interacts with LSD1 and EZH2 and suppresses CDKN1A (P21) and E-cadherin transcription	74
DNA methylation	SNHG1	up	Promotes DNMT1 expression	36
	MEG3	down	DNA methylation affects the expression of MEG3 and its targeting miR-181a-5p	39
	HOTTIP	up	Increases HoxA13 expression by down-regulating DNA methylation at the E1 site	75
	LINC00673	up	Suppresses KLF4 expression by interacting with EZH2 and DNMT1	76
	Linc00441	up	Recruits DNMT1 to the RB1 promoter and suppresses RB1 expression	77
mRNA translation	MACC1-AS1	up	Stabilizes MACC1 mRNA	41
	GMAN	up	Promotes translation of ephrin A1 messenger RNA	44
	KRT7-AS	up	Stabilizes KRT7 mRNA	78
CeRNA	KCNQ1OT1	down	KCNQ1OT1/miR-9/LMX1A	50
	LINC00682	down	LINC00682/miR-9/LMX1A	51
	COL1A1-014	up	COL1A1-014/miR-1273h-5p/CXCL12	52
	MYOSLID	up	MYOSLID/miR-29c-3p/MCL-1	53
	CTC-497E21.4	up	CTC‑497E21.4/miR-22/NET1	57
	LINC00346	up	LINC00346/miR-34a-5p/CD44, NOTCH1, AXL	79
	UCA1	up	UCA1/miR-26a/b, miR-193a, miR-214/PDL1	80
	LINC01133	down	LINC01133/miR-106a-3p/APC	81
	MT1JP	down	MT1JP/miR-92a-3p/FBXW7	82
	LINC01939	down	LINC01939/miR-17-5p/EGR2	83
	H19	up	H19/miR-22-3p/Snail	84
	UCA1	up	UCA1/miR-203/ZEB2	85
	HNF1A-AS1	up	HNF1A-AS1/miR-661/CDC34	86
	UFC1	up	UFC1/miR-498/Lin28b	87
	GCRL1	up	GCRL1/miR-885-3p/CDK4	88
	SMARCC2	down	SMARCC2/miR-551b-3p/TMPRSS4	89
	BC032469	up	BC032469/miR-1207-5p/hTERT	90
LncRNAs act on proteins	MIR4435-2HG	up	Binds to DSP and inhibits its expression	60
	KRT19P3	down	Enhances COPS7A protein stability in GC cells	62
	LINC00707	up	Interacts with mRNA stabilizing protein HuR	65
	HOXC-AS3	up	Binds to YBX1	69
	SNHG12	up	Increases the phosphorylation of PI3K by directly binding to it	91
	SEMA3B-AS1	down	Interacts with MLL4	92
	pancEts-1	up	Directly interacts with NONO to increase its interaction with ERG	93
	GALNT5 uaRNA	up	Binds with HSP90	94
	HOTAIR	up	Interacts with Mex3b	95

**Table 2:** The relationships between lncRNAs and the hallmarks of gastric cancer

Function	LncRNA	Expression	Target	Reference
LncRNAs and proliferation	Linc00152	up	EGFR	99
	AK123072	up	EGFR	100
	ZFPM2-AS1	up	MIF/P53	104
	OnclncRNA-626	up	SRSF1/p53	107
	GAS5	up	eIF4A3/P53	147
	PWRN1	down	miR-425-5p/PTEN/Akt/MDM2/p53	148
	AK023391	up	PI3K/Akt/p53	149
	TUBA4B	down	miR-214 and miR-216a/b /PTEN	109
	LINC00470	up	METTL3/PTEN	110
	PCAT-1	up	EZH2/PTEN	150
	Linc00441	up	RB1	77
	MEG3	up	P53,RB	151
	BC032469	up	BC032469/miR-1207-5p/hTERT	90
LncRNAs and resistance to cell death	XIAP-AS1	up	XIAP	118
	TINCR	up	KLF2	121
	LINC01419	up	PI3K/Akt1/mTOR	124
	HAGLROS	up	miR-100-5p/mTOR ,mTORC	152
LncRNAs and metastasis	MAGI2-AS3	up	MAGI2-AS3/miR-141/200a/ZEB1	128
	DLX6-AS1	up	miR-204-5p/OCT1	130
	HULC	up	miR-9-5p/MYH9	153
	DLX6-AS1	up	FUS-regulated MAP4K1	154
	SNHG16	up	DKK3	155
	ELIT-1	up	TGFβ/Smad3 signaling	156
	LINC01133	up	miR-106a-3p/APC	81
	MEG3	down	E-cadherin,Vimentin	157
	LncRNA-ATB	up	MiR‐141‐3p/TGFβ2	158
	LINC00675	down	vimentin	159
	SNAI1	up	CDH1	160
LncRNAs and DNA damage	PANDAR	up	NFYA, p53/CDKN1A	134
	NORAD	up	miR-608	136
LncRNAs and angiogenesis	PVT1	up	STAT3/VEGFA	139
	LINC01314	down	KLK4	140
	ZEB2-AS1	up	Wnt/β-catenin pathway	161
	MEG3	down	MEG3/miR-21	162
LncRNAs and the immune response	UCA1	up	miR-193a, miR-214/PDL1	80
LncRNAs and the stemness of GC	LOXL1-AS1	up	LOXL1-AS1/miR-708-5p/SOX2	145
	SPRY4-IT1	up	SPRY4-IT1/miR-101-3p/AMPK	146
	MACC1-AS1	up	MACC1-AS1/miR-145-5p	163
	MALAT1	up	SOX2 mRNA	164

**Table 3:** LncRNAs as diagnostic biomarkers in gastric cancer

Clinical application	LncRNA	Expression	Diagnosis accuracy	Reference
Diagnostic biomarker	H19	up	AUC was 0.838; p<0.001; sensitivity, 82.9%; specificity, 72.9%	171
	FAM49B-AS, GUSBP11 and CTDHUT	up	The combined area under curve of these lncRNAs was 0.818	172
	lnc-GNAQ-6:1	down	AUC was 0.732	175
	UCA1	up	AUC was 0.759; The sensitivity and specificity were 93.20% and 78.63%	176
	B3GALT5-AS1	up	AUC was 0.816	177
	HOTAIR	up	AUC was 0.8416; The sensitivity and specificity were 66.67 and 87.04%	178
	lncUEGC1	up	AUC was 0.8406	179
	PANDAR, FOXD2-AS1, and SMARCC2	up	The AUC of the combinative diagnostic value of these three lncRNAs was 0.839	180
	TINCR, CCAT2, AOC4P, BANCR, and LINC00857	up	The AUC of the combinative diagnostic value of these five lncRNAs was 0.91	181

**Table 4:** LncRNAs as prognostic biomarkers in gastric cancer

Clinical application	LncRNA	Expression	Clinical significance	Reference
Prognostic biomarker	CASC19	up	Higher pathologic TNM stage, pathologic T stage, lymph node metastasis, and poor overall survival	173
	SNHG6	up	Invasion depth, lymph node metastasis, distant metastasis and TNM stage	182
	MIR100HG	up	TNM stage, tumor invasion, lymph node metastasis, and distant metastasis	183
	Sox2ot	up	TNM stage, tumor depth, lymph node metastasis, and distant metastasis	184
	MALAT1	up	Distant metastasis	185
	NEAT1	up	Clinical stage, histological type, lymph node metastasis, and distant metastasis	174
	SNHG8	up	Predicts poor prognosis, shorter survival time	186
	CTD-2510F5.4		Pathological grade, vascular or nerve invasion, AJCC TNM stage and OS, shorter MST	187

**Table 5:** LncRNAs and the resistance to therapy

Clinical application	LncRNA	Expression	Pathway	Reference
chemoradiotherapy resistance	CRAL	down	miR-505/CYLD/ AKT	188
	ARHGAP5-AS1	up	ARHGAP5	189
	PCAT1	up	EZH2/PTEN	192
	MALAT1	up	miR-23b-3p /ATG12	193
	HULC	up	FoxM1	194
	D63785	up	miR-422a/MEF2D	195

## Abbreviations

AGC: advanced GC
ASO: antisense oligonucleotide
ATG4B: autophagy-associated 4B
AUC: area under the curve
BTG2: B-cell translocation gene 2
CA: carbohydrate antigen
CCNE2: cyclin E2
CDK: cyclin-dependent kinase
CDK16: cyclin‑dependent kinase 16
CEA: carcinoembryonic antigen
CRT: chemoradiotherapy
CSC: cancer stem cell
CXCL12: chemokine (CXCmotif) ligand
DDR: DNA damage response
DSP: desmoplakin
ECM: extracellular matrix
EGFR: epidermal growth factor receptor
EMT: epithelial-to-mesenchymal transition
eRNA: enhancer-associated RNA
FUS: fused in sarcoma
GC: gastric cancer
GEF: guanine exchange factor
GMAN: gastric cancer metastasis associated long noncoding RNA
GMAN-AS: antisense GMAN RNA
HDAC: histone deacetylase
hTERT: telomerase reverse transcriptase
H3K27me3: trimethylation of lysine residue 27 of histone 3
H3K4: histone H3 lysine K4
JAM-A: junctional adhesion molecule A
JMJD3: jumonji-domain containing 3
KLF: kruppel-like factor
KRT19P3: Keratin 19 Pseudogene 3
LIM-HD: LIM-homedomain
LMX1A: Lim homeobox transcription factor 1
lncRNA: long non-coding RNA
LOXL1-AS1: LOXL1 antisense RNA 1
MACC1: metastasis-associated in colon cancer-1
MACC1-AS1: MACC1 antisense RNA 1
MED18: mediator subunit 18
MMP: member matrix metalloproteinase
MNX1-AS1: MNX1 antisense RNA 1
MRE: miRNA response element
MSS: microsatellite stablity
mTOR: rapamycin
m6A: N6-methyladenosine
ncRNA: non-coding RNA
NET1: neuroepithelial cell transforming gene 1
NFYA: nuclear transcription factor Y subunit alpha
NF-κB: nuclear factor kappa-B inhibitor
NORAD: The noncoding RNA activated by DNA damage
PANDAR: promoter of CDKN1A antisense DNA damage activated RNA
PCA3: PC antigen 3
PCD: programmed cell death
p-PI3K: phosphoinositide 3-kinase
PRC2: polycomb repressive complex
PROMPT: promoter upstream transcript
RBP: RNA-binding protein
RHOA: Ras homolog gene family member A
RIP: RNA immunoprecipitation
ROC: receiver operating characteristic curve
RTK: receptor tyrosine kinases
siRNA: small interference RNA
SMD: STAU1 mediated mRNA degradation
SNCG: γ-synuclein
SNHG1: small nucleolar RNA host gene 1
SNHG3: small nucleolar RNA host gene 3
SPG: serum pepsinogen
SPRY1: sprouty RTK signaling antagonist 1
SRSF1: serine- and arginine-rich splicing factor 1
TEAD: TEA domain
TNF: tumor necrosis factor
TRAIL: tumor necrosis factor (TNF) related apoptosis inducing ligand
TSS: transcription start site
UCA1: urothelial cancer-associated 1
XIAP: X-linked apoptosis inhibitor
YBX1: Y-box binding protein 1
YTHDF2: YTH domain family 2
